# Prevalence of *Coxiella burnetii *in clinically healthy German sheep flocks

**DOI:** 10.1186/1756-0500-5-152

**Published:** 2012-03-19

**Authors:** Angela Hilbert, Gernot Schmoock, Hannah Lenzko, Udo Moog, Roland Diller, Andreas Fröhlich, Lothar Hoffmann, Steffen Horner, Michael Elschner, Herbert Tomaso, Klaus Henning, Heinrich Neubauer, Lisa D Sprague

**Affiliations:** 1 Institut für Epidemiologie, Friedrich-Loeffler-Institut, National Reference Laboratory for Q-fever, Wusterhausen, Germany; 2 Institut für Bakterielle Infektionen und Zoonosen, Friedrich-Loeffler-Institut, Jena, Germany; 3 Institut für Molekulare Pathogenese, Friedrich-Loeffler-Institut, Jena, Germany; 4 Tiergesundheitsdienst, Thüringer Tierseuchenkasse, Jena, Germany; 5 Thüringer Landesamt für Lebensmittelsicherheit und Verbraucherschutz, Bad Langensalza, Germany; 6 Thüringer Ministerium für Soziales, Familie und Gesundheit, Erfurt, Germany

**Keywords:** *Coxiella (C.) burnetii*, Zoonosis, Sheep, Prevalence

## Abstract

**Background:**

Current epidemiological data on the situation of *Coxiella (C.) burnetii *infections in sheep are missing, making risk assessment and the implementation of counteractive measures difficult. Using the German state of Thuringia as a model example, the estimated sero-, and antigen prevalence of *C. burnetii *(10% and 25%, respectively) was assessed at flock level in 39/252 randomly selected clinically healthy sheep flocks with more than 100 ewes and unknown abortion rate.

**Results:**

The CHECKIT™ Q-fever Test Kit identified 11 (28%) antibody positive herds, whereas real-time PCR revealed the presence of *C. burnetii *DNA in 2 (5%) of the flocks. Multiple-locus variable number of tandem repeats analysis of 9 isolates obtained from one flock revealed identical profiles. All isolates contained the plasmid QpH1.

**Conclusions:**

The results demonstrate that *C. burnetii *is present in clinically inconspicuous sheep flocks and sporadic flare-ups do occur as the notifications to the German animal disease reporting system show. Although *C. burnetii *infections are not a primary veterinary concern due to the lack of significant clinical impact on animal health (with the exception of goats), the eminent zoonotic risk for humans should not be underestimated. Therefore, strategies combining the interests of public and veterinary public health should include monitoring of flocks, the identification and culling of shedders as well as the administration of protective vaccines.

## Background

*C. burnetii *is an obligate intracellular bacterial pathogen and the causative agent of Q- fever, a worldwide occurring zoonosis, and notifiable disease in many countries including Germany. The organism is very resistant and can persist in the environment in a spore-like state for weeks; once airborne it can be transported long distances by the wind [1-3]. Numerous species including dogs, cats, birds, arthropods, and wildlife can harbour the agent, however, cattle, sheep, and goats are considered to be the main reservoir [4]. Infection in animals is mostly subclinical or inapparent but can occasionally lead to abortions or birth of weak offspring. During parturition, large numbers of the organism are shed into the birth fluids, but smaller amounts can also be found in milk, faeces, and urine [5]. Transmission to humans occurs mainly via inhalation of fomites, seldom through ingestion of contaminated raw milk, and very rarely via person-to-person contact [4]. In humans, disease ranges from asymptomatic to severe and can be fatal. The clinical picture presents itself with fever or influenza-like illness. Pneumonia, hepatitis, meningoencephalitis, myocarditis, and pericarditis can occur as life-threatening complications. Infection in early pregnancy can lead to abortion and in later stages of pregnancy to premature labour [6].

According to the Federal Statistical Office, there are approx. 2.35 million sheep in Germany. Despite the gradual decline in the German sheep population and falls in the price of wool over the past years, foodstuffs obtained from sheep (i.e. meat and milk) are enjoying an increase in popularity. In the state of Thuringia, the stock of sheep amounts to nearly 190.000 and serves not only as a source of meat and milk, but also plays an important role in landscape management and nature conservation. The sheep are distributed among approx. 6300 flocks of which 252 contain more than 100 ewes. Although several German studies describing the seroprevalence of *C. burnetii *in sheep during outbreaks of Q-fever exist [7-9], no current prevalence data are available. Moreover, seroprevalence studies in asymptomatic, i.e. clinically healthy flocks, and in flocks with prevailing infections are missing, making risk assessment and the implementation of counteractive measures and regulations difficult.

Accordingly, the aim of this study was to estimate the sero- and antigen prevalence of *C. burnetii *at flock level among clinically healthy non-vaccinated sheep flocks using the state of Thuringia as a model example.

## Results

### Serology

Based on the sensitivity and specificity of the used test of 100% a flock was considered sero-positive if at least one animal tested positive in the ELISA. Of the 39 evaluated flocks with more than 100 ewes, 11 were serologically positive (28%; Table 1). The exact 95% confidence interval for the flock-level prevalence was estimated as 15-45%.

**Table 1:** Summary of flocks, collected -, positive serum-, and DNA (PCR positive) samples

			# positive samples vs. # of samples taken					
			
flock #	flock size (ewes)	abortion rate (%)^§^	serum		vaginal swab	rectal swab	afterbirth	foetus/foetal swab
1	500	0	**1/28**	0/11	0/11	0/11	-	-
2	1000	0	0/29	0/11	0/11	0/11	0/9	-
3	1800	< 1	**4/29**	**2/11**	0/11	0/11	0/11	-
4	1300	0	0/29	0/11	0/11	0/11	-	-
5	1200	3	**4/29**	**3/11**	0/11	0/11	0/3	0/4
6	500	0	0/29	0/11	0/11	0/11	-	-
7	450	0	0/29	0/11	0/11	0/11	0/9	-
8	1100	< 1	**3/30**	**1/11**	0/11	0/11	-	-
9	1800	0	**1/29**	0/11	0/11	0/11	-	-
10	550	0	**4/29**	**2/11**	0/11	0/11	0/3	-
11	700	< 1	**2/29**	**2/11**	0/11	0/11	-	-
12	330	0	0/30	0/11	0/11	0/11	0/2	-
13*	2500	2	**6/30**	**1/11**	**11/11**	**11/11**	7/11**	-
14	400	< 1	0/29	0/11	0/11	0/11	-	-
15	700	< 1	0/29	0/11	0/11	0/11	-	-
16	750	< 1	0/29	0/11	0/11	0/11	-	-
17	900	< 1	0/29	0/11	0/11	0/11	0/3	0/2
18	250	< 1	0/29	0/11	0/11	0/11	-	-
19	450	< 1	0/29	0/11	0/11	0/11	0/1	-
20	1000	< 1	0/29	0/11	0/11	0/11	-	-
21	500	< 1	0/29	0/11	0/11	0/11	-	-
22	406	< 1	0/29	0/11	0/11	0/11	-	-
23	700	< 1	**3/29**	**2/11**	0/11	0/11	-	-
24	700	0	0/29	0/11	0/11	0/11	-	-
25	550	< 1	**5/29**	**1/11**	0/11	0/11	-	-
26	1000	1	0/29	0/11	0/11	0/11	-	-
27	317	< 1	0/29	0/11	0/11	0/11	-	-
28	350	6	0/29	0/11	0/11	0/11	0/1	-
29	420	0	0/29	0/11	0/11	0/11	-	-
30	1178	< 1	0/29	0/11	0/11	0/11	-	-
31	500	< 1	0/29	0/11	0/11	0/11	-	-
32	120	0	0/29	0/11	0/11	0/11	0/4	-
33	115	< 1	**17/29**	**6/11**	**1/11**	**1/11**	-	-
34	400	< 1	0/29	0/11	0/11	0/11	0/1	-
35	650	< 1	0/29	0/11	0/11	0/11	-	-
36	133	0	0/29	0/11	0/11	0/11	-	-
37	377	< 1	0/29	0/11	0/11	0/11	0/2	-
38	409	< 1	0/29	0/11	0/11	0/11	-	-
39^&^	25	0	25		11	11	-	-
40	460	< 1	0/29	0/11	0/11	0/11	-	-

**total**			**1158**		**440**	**440**	**71**	**6**

^§^according to farmers and Thuringian sheep health authority; *history of *C. burnetii *infection; **7/11 DNA positive, from 3/7 isolation of *C. burnetii; *- no samples; ^&^excluded from study

### Isolation of *C. burnetii*

*C. burnetii *was isolated and propagated from nine afterbirths acquired from flock 13. Three of these isolates were obtained from samples collected during the prevalence study in May 2009 (Table 1), one isolate was obtained from a sample collected before initiation of the study, and the remaining five isolates originated from additional samples collected in June 2009 (Table 2).

**Table 2:** Additional samples collected in flocks 3, 13 and 33 (analysed by ELISA or PCR)

			# positive samples vs. # of samples taken				
			
flock # date	flock size (ewes)	abortion rate (%)	serum	vaginal swab	rectal swab	afterbirth	foetus/foetal swab
**3 **(01/2010)	1800	< 1	1/34	0/11	0/11	0/5	0/8
**13**							
(07/2005)		-	-	1/51	-	-	-
(12/2005)		-	-	-	-	-	1/14
(01/2008)	2500	-	-	-	-	0/18	-
(04/2008)		2	-	-	-	2/34*	-
(05/2009)		-	1/6	-	-	8/19*	0/1
(06/2009)		-	-	-	-	18/24*	-
(07/2010)		-	-	-	-	0/58	-
(03/2011)		-	-	0/107	-	-	-
**33**							
(02/2010)	115	< 1	42/89	5/26	-	-	-
(07/2010)		-	-	-	-	0/11	-

- no samples; *1/34 *C. burnetii *isolate; *1/19 *C. burnetii *isolate; *4/24 *C. burnetii *isolates

### Detection of *C. burnetii *by means of PCR

Based on the specificity and sensitivity of the PCR assay of 100% a flock was considered antigen-positive if at least one animal tested positive in the PCR. *C. burnetii *DNA was detected in two flocks (5%; Table 1). The exact 95% confidence interval for the flock-level prevalence was estimated as 0.6-17%.

### Genotyping of *C. burnetii *by means of MLVA and plasmid type determination

**Figure 1:**
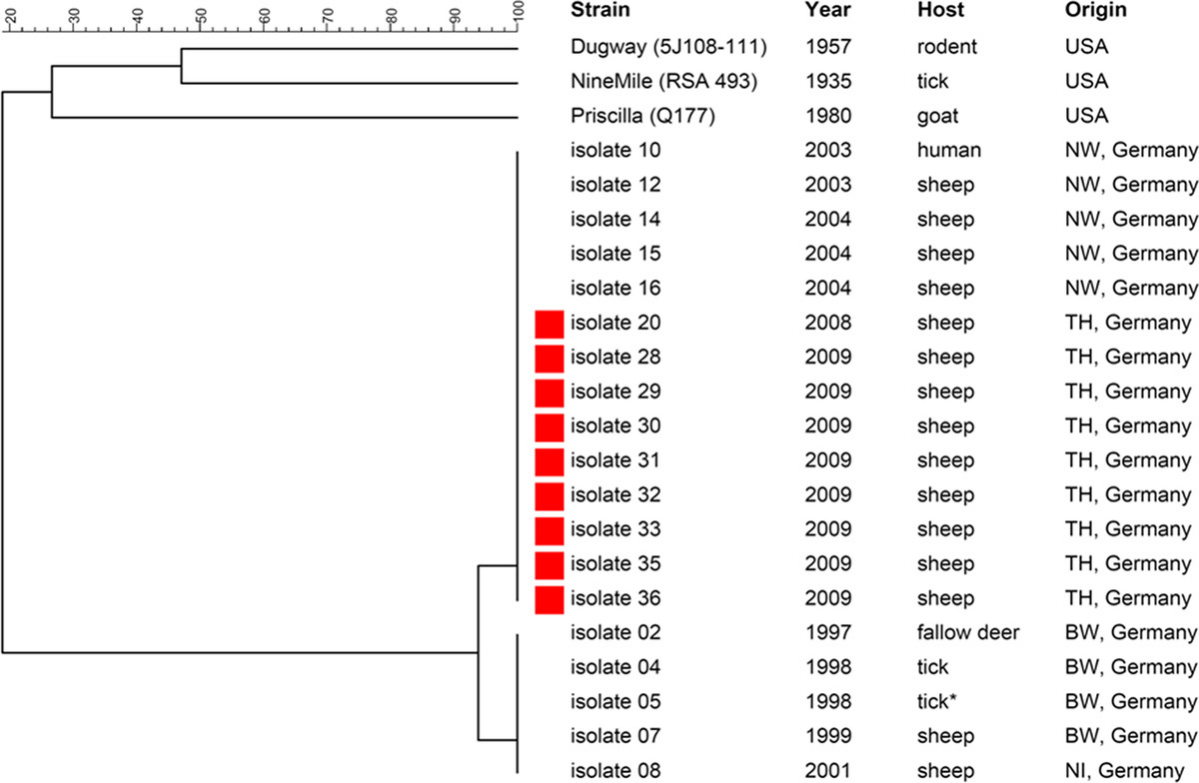
**Phylogenetic tree of German *C. burnetii *isolates on the basis of 17 multilocus variable number of tandem repeat analyses (MLVA)**.

In order to investigate the genetic relationship among the isolates obtained from afterbirths collected in flock 13, MLVA were done. These revealed identical VNTR profiles all clustering into the same group (Figure 1). All tested isolates were shown to contain the plasmid QpH1.

## Discussion

Epidemiological data regarding the distribution of *C. burnetii *in sheep in Germany are scarce and based on data obtained during Q-fever outbreaks [7,8] and on materials submitted for routine laboratory examination [9]. Data describing the epidemiological situation in clinically inconspicuous flocks and between outbreaks are missing. The present study, therefore, aimed at estimating the sero- and antigen prevalence of *C. burnetii *in randomly chosen non-vaccinated sheep flocks throughout the state of Thuringia with unknown abortion status.

Our study revealed that 28% of the tested flocks were serologically positive. Other studies assessing the seroprevalence of *C. burnetii *in sheep found rates ranging between 1% to 47% in Germany [8-10], 3% to 22% in Turkey [11,12], 12% in northern Spain and 31.7% in Gran Canaria [13,14], 11.8% in southern Italy [15], and up to 73% in Bulgaria [16]. However, the direct comparison of our results with the prevalences found in the above listed studies is problematic, due to considerable differences in study design and evaluation methods (prevalence in single animals vs. flock prevalence), flock size, flock management, abortion rate, number of samples tested and the detection methods applied (CFT, IFAT, competitive ELISA).

We next compared our data with the results obtained from the ovine samples (> 1500) submitted to the German National Reference Laboratory (NRL) for Q-fever. Evaluation of the samples sent to the NRL between August 2007 and July 2010 determined a seroprevalence of 10.8%, which is in agreement with our estimated prevalence value; yet one has to bear in mind that the evaluation of these samples is biased. We also compared our results with those acquired from the contemporaneous Thuringian *Brucella *screening in which the ovine samples were additionally tested for the presence of *C. burnetii *antibodies with the CHEKIT™ Q-fever Test Kit. The screening revealed a seroprevalence of 31% (4/13 flocks) when evaluating the results from the flocks with > 100 ewes which is also in agreement with our results (data not shown). We can not rule out a possible lower sensitivity of the used ELISA due to the fact that it does not use ruminant antigen. However, this particular ELISA is the only one on the "List of certified products pursuant to section 17c Animal Diseases Act" in Germany. Moreover, even if we had found more positive animals within a flock, it would not have had an influence on the flock prevalence. Further positive flocks on the other hand, would have altered the flock prevalence.

Despite the fact that serological screening to test for antibodies against *C. burnetii *is carried out on a regular basis, results should be interpreted with caution. Recent evaluation studies on serum samples obtained from cattle, sheep and goats showed that shedding animals are not always reliably detected; single animals may seroconvert but not shed the agent, whereas others shed the agent without or with delayed production of antibodies [17-19]. One also has to bear in mind that antibodies may continue to circulate long after the agent has been cleared from the organism [20].

Our PCR analyses detected *C. burnetii *DNA in 5% of the flocks assessed in the study. Recent PCR-based studies identified *C. burnetii *in 9% of tested sheep flocks in northern Spain [21] and in 18.6% of farms with small ruminants in southern Italy [22]. Two further studies on samples obtained from either ovine abortions in Sardinia [23] or ovine foetal organ samples and placentae in Portugal [24] discovered *C. burnetii *in 10.9% and in 36% of the cases, respectively. A Turkish study assessing milk samples collected from 22 flocks determined 6.5% *C. burnetii *positive animals in 12 flocks with a history of abortion and no positives in flocks without a history of abortion [25]. But again, comparison of the data is difficult due to the differences in study design, sampling, and methods applied. However, the evaluation of the *C. burnetii*-tested samples of the contemporaneous Thuringian *Brucella *screening using different sampling criteria, e.g. health status, flock size, etc. revealed an antigen prevalence of 25%, which is in agreement with our estimated prevalence.

Although we found eleven seropositive flocks, only two of the flocks (13, 33) were DNA-positive. Nonetheless, we were able to obtain nine isolates from flock 13 in afterbirths collected between April 2008 and June 2009. All isolates were genetically identical as shown by MLVA and determination of the plasmid type. The QpH1 plasmid, first isolated from a tick [26], has been regularly found in isolates obtained from cattle, sheep, and goats [27]. We were intrigued to find that our nine isolates clustered into the same group as the isolates obtained from sheep and a human isolate linked to a Q-fever outbreak back in 2003 in North Rhine Westphalia [28]. Our findings argue for the circulation of a particular *C. burnetii *strain infecting both man and animal in central Germany, however, more isolates must be tested to corroborate this hypothesis. We did not observe any genetic variations as described in the exceptional Dutch outbreak (2007-2010) [29,30] in our comparatively small panel, although we used more loci and purified DNA from isolates. It is worthwhile mentioning that *C. burnetii *was already detected back in 2005 in flock 13, indicating the persistence of infection in this flock. However, since no isolates were obtained in 2005 we can not confirm the circulation of one particular strain or exclude re-introduction.

Identification of shedders is central to any eradication or surveillance programme. We believe that monitoring of clinically inconspicuous sheep flocks for the presence of *C. burnetii *infection can be reliably done by analysing the afterbirths. As shown in Table 2, in January 2008, none of the examined afterbirths reacted positive in the PCR assays but by June 2009, 75% of the tested afterbirths were positive. Samples obtained from afterbirths and vaginal swabs taken between July 2010 and March 2011 were again negative. Our observations are in agreement with the findings of others, describing that shedding is not a continuous process [31,32]. However, it is also possible that the amounts of shed or circulating bacteria might not suffice to maintain an infection cycle. The threshold level of bacteria required to produce a clinically apparent infection in an animal and to what extent virulence of the circulating strain affects infection and clinical presentation are still unknown. Studies assessing possible individual or breed related immunity are also missing.

## Conclusions

Based on the assumed prevalence at flock level, we were able to demonstrate that *C. burnetii *is present in clinically inconspicuous sheep flocks. Although *C. burnetii *infections are not a primary veterinary concern, due to the lack of significant impact on animal health, the zoonotic risk for humans should not be underestimated. Therefore, strategies combining the interests of public and veterinary public health should include the identification and culling of shedders as well as the implementation of protective vaccines.

## Materials and methods

### Sheep flocks and sampling procedures

The present study was designed as a cross sectional study. Forty unvaccinated flocks (Table 1) distributed throughout the state of Thuringia with more than 100 ewes and an unknown abortion rate, were chosen at random from the given population of 252 flocks. Flocks were kept on pasture, lambing took place in-doors. Based on the reported cases to the German animal disease reporting system, the sample size was calculated as such that with an assumed minimum seroprevalence of 10% and an antigen prevalence of 25% within the flock, at least one infected animal would be detected with 95% confidence under the assumption of 100% sensitivity and specificity of the diagnostic test used [33].

Sera and swabs were collected at random during the lambing seasons by and under the supervision of a veterinarian (UM) from the Thuringian sheep health service during his regular flock management visits between February 2009 to June 2009 (flocks 1-13) and December 2009 to April 2010 (flocks 14-40). In practice, the sampling was carried out as follows: Serum samples were collected from 29 ewes per flock on day 1 or day 2 post partum. For cultivation and PCR assays, one vaginal and one rectal swab were obtained from 11/29 ewes. Afterbirths and foetuses were collected when available. Of the 1281 serum samples, 477 vaginal swabs, 451 rectal swabs, 188 afterbirths and 15 foetuses/foetal/pharyngeal swabs collected, a total of 1158 serum samples, 440 vaginal swabs, 440 rectal swabs, 71 afterbirths and 6 foetuses/foetal swabs from 39 flocks were evaluated (Table 1). Since *Coxiella *and *Chlamydia *spp screening is part of the Thuringian flock management system no ethics approval and consent was necessary [34].

Additional samples (not included in the study) were obtained from flocks 3 (January 2010), 13 (January and April 2008, May and June 2009, and July 2010) and 33 (July 2010) (Table 2).

### Sample preparation and conservation

Blood was drawn from the jugular vein with a 14 gauge needle into 7.5 mL serum Monovettes (Kabe GmbH, Nümbrecht-Elsenroth, Germany) and stored upright at RT for 12 h. The Monovettes were then centrifuged at 1500 ×*g *for 10 min, the supernatant serum removed and stored at -20°C until further use. Swabs collected for nucleic acid extraction were transferred to 200 μL lysis buffer [(6 M guanidiumisothiocyanate, 10 mM urea, 20% (v/v) Triton X-100 and 10 mM Tris HCl (pH 4.4))] (Roche Diagnostics, Mannheim, Germany) and stored at 4°C. Afterbirths and organ samples from aborted foetuses were stored at -80°C until further use.

### ELISA

Serum samples were tested for the presence of antibodies to *C. burnetii *using the CHECKIT™ Q-fever Test Kit (Idexx GmbH, Liebefeld-Bern, Switzerland) according to the manufacturer's instructions. All measurements were performed in duplicate. Results were normalised using the positive and negative control sera provided in the kit and expressed as percentage of the positive control according to the following formula: [(OD sample - OD negative control)/(OD positive control - OD negative control)] × 100. Sera with values below 30% were considered negative, sera with values between 30 and 40% were considered inconclusive, and sera with values greater 40% were considered as positive.

### Cell culture

Propagation and isolation of *C. burnetii *was performed using Buffalo Green Monkey (BGM) cells in UltraCulture medium (Bio Whittaker, Walkersville, USA) supplemented with 1% non essential amino acids (100×), 1% vitamins, and 2 mmol L-glutamine (all Biochrom, Berlin, Germany). Cells were seeded into 25 cm^2 ^tissue culture flasks (Greiner Bio-One GmbH, Frickenhausen, Germany) and maintained in a humidified atmosphere with 5% CO_2 _at 37°C. The cell monolayers were assessed for confluent growth on the day of inoculation.

Vaginal swabs were rehydrated in 2000 μL of Hank's medium, centrifuged at 15 000 ×*g *for 10 min at RT, and the resulting pellet resuspended in 1000 μL Hank's medium (Biochrom). Organ samples (approx. 10 g) were mechanically disrupted, homogenised and resuspended in 40 mL Hank's medium. This solution was subsequently filtered through 0.45 - 0.2 μm syringe filters (Minisart, Sartorius, Hannover, Germany). Between 100 μL - 500 μL of the resuspended pellet or the final filtrate was used for inoculation. After 12-24 h, the medium was replaced by fresh UltraCulture medium, also used for further propagation. Cell cultures were monitored weekly by phase contrast microscopy and propagated for up to six months. Specimens that showed intracellular growth of microorganisms were stained according to the method of Giménez [35]. Cultures were regarded as positive when small red inclusions containing coccoid rods were observed.

### DNA extraction from samples

DNA from vaginal, rectal, and foetal swabs as well as from foetal organs was isolated using the High Pure PCR Template Preparation Kit™ (Roche Diagnostics) according to the manufacturer's instructions. Organ samples were cut into 50 mg sections, mechanically disrupted and digested over night in 200 μL lysis buffer with 40 μL proteinase K (20 mg/mL) (Roche Diagnostics) at 37°C.

### Polymerase chain reaction (PCR)

#### Conventional PCR

In order to avoid abortive cell cultivation, samples for cultivation were tested beforehand at the National Reference Laboratory for Q-fever in Wusterhausen with a nested PCR method targeting the *com 1 *gene encoding a 27 kDa outer membrane protein of *C. burnetii *[36]. Conventional PCR was carried out in a TC-412 Thermocycler (Techne AG, Burkhardtsdorf, Germany). From each PCR reaction, 15 μL were analysed by agarose gel electrophoresis (1.5% w/v in Tris Borate EDTA buffer).

### Real-time PCR

Detection of *C. burnetii *was performed with a TaqMan based real-time PCR assay targeting the transposase element IS1111 as described by Klee et al. [37] using a Stratagene Mx3000P Thermocycler (Agilent Technologies, Santa Clara, CA, USA). Tenfold serial dilution of cloned IS1111 gene fragments ranging from 1 × 10^0 ^to 1 × 10^5 ^plasmid copy numbers were added for *Coxiella *DNA quantification and sensitivity control of the assay. The cycle threshold value (Ct) was calculated by the instrument's software MxPro3000P v 4.01. A negative result was assigned when no amplification occurred or when the cycle threshold value was ≥ 40.

#### Identification of the plasmid type in the *C. burnetii *isolates

Identification of the plasmid type of the isolates was done according to a modified procedure described by Zhang et al. [36]. PCR assays were conducted on a MasterCycler ep Thermocycler (Eppendorf, Germany). From each PCR reaction, 5 μL were analysed by agarose gel electrophoresis (1.5% w/v in Tris Borate EDTA buffer).

### Genotyping of *C. burnetii *isolates by means of multiple-loci variable number of tandem repeats analysis (MLVA)

Genotyping of the 9 *C. burnetii *isolates obtained from flock 13 was done according to the VNTR method described by Arricau-Bouvery et al. [32] using 17 markers. PCR assays were conducted on a MasterCycler ep Thermocycler (Eppendorf, Germany). Compatible primer pairs were subsequently multiplexed and the forward primer for each pair labelled at the 5'-end with a fluorescent dye (dye set G5, Applied Biosystems). The PCR products were pooled according to their group and diluted 1:100 in LiChrosol water (VWR International, Germany). One μL of this solution was mixed with 13.7 μL Hi-Di formamide and 0.3 μL GeneScan™ 1200 LIZ Size Standard (both Applied Biosystems) for the reproducible sizing of the fragments, denatured for 3 min at 93°C, and cooled on ice. The PCR products were separated in an ABI 3130 Genetic Analyzer (Applied Biosystems) using a 36 cm array and POP7 polymer. Data obtained from the PeakScanner Ver.1.0 (Applied Biosystems) were analysed with the Bionumerics 6.0 software package (Applied Maths). The clustering analysis was based on the categorical coefficient and unweighted pair group method using arithmetic averages (UGPMA).

## Competing interests

The authors declare that they have no competing interests.

## Authors' contributions

AH: processed the samples for ELISA and cultivation and evaluated the data. GS: processed the DNA samples for PCR and MLVA and evaluated the data. HL: collected the samples. UM: collected the samples and helped with the contacting of the sheep farmers. RD: designed the study. AF: carried out additional statistics. LH and SH: evaluated the samples from the Thuringian Brucellosis screening for the presence of *C. burnetii*. ME: contributed to the study design. HT: helped with editing the manuscript. KH: helped with the ELISA and cultivation of the *C. burnetii *isolates. HN: contributed to the study design, obtained the funding, contributed to the interpretation of the data, and helped with editing and revision of the manuscript; LDS: contributed to the study design, evaluated the data, drafted, and wrote the manuscript. All authors read and approved the final manuscript.

## References

[B1] Tissot-Dupont H Amadei MA Nezri M Raoult D Wind in November, Q-fever in December Emerg Infect Dis 2004 10 1264 1269 15324547 10.3201/eid1007.030724 PMC3323349

[B2] Gilsdorf A Kroh C Grimm S Jensen E Wagner-Wiening C Alpers K Large Q-fever outbreak due to sheep farming near residential areas, Germany, 2005 Epidemiol Infect 2008 136 1084 1087 17892631 10.1017/S0950268807009533 PMC2870892

[B3] Wallensten A Moore P Webster H Johnson C van der Burgt G Pritchard G Ellis-Iversen J Oliver I Q-fever outbreak in Cheltenham, United Kingdom, in 2007 and the use of dispersion modelling to investigate the possibility of airborne spread Euro Surveill 2010 15 12 19521 20350497

[B4] Maurin M Raoult D Q fever Clin Microbiol Rev 1999 12 518 553 10515901 10.1128/cmr.12.4.518 PMC88923

[B5] Rodolakis A Q-fever in dairy animals Ann N Y Acad Sci 2009 1166 90 93 10.1111/j.1749-6632.2009.04511.x 19538267

[B6] Carcopino X Raoult D Bretelle F Boubli L Stein A Q-fever during pregnancy: a cause of poor fetal and maternal outcome Ann N Y Acad Sci 2009 1166 79 89 10.1111/j.1749-6632.2009.04519.x 19538266

[B7] Lange S Klaus G Seroepidemiological studies on the detection of Q fever in sheep in middle Thuringia Berl Munch Tierarztl Wochenschr 1992 105 333 335 1463436

[B8] Lange S Söllner H Dittmar H Hofmann J Lange A Q fever antibody titre-follow-up study in cattle with special reference to pregnancy Berl Munch Tierarztl Wochenschr 1992 105 260 263 1524578

[B9] Sting R Breitling N Oehme R Kimmig P The occurrence of *Coxiella burneti *in sheep and ticks of the genus Dermacentor in Baden-Württemberg Dtsch Tierarztl Wochenschr 2004 111 390 394 15568636

[B10] Hellenbrand W Breuer T Petersen L Changing epidemiology of Q fever in Germany, 1947-1999 Emerg Infect Dis 2001 7 789 796 10.3201/eid0705.010504 11747689 PMC2631891

[B11] Kilic S Pasa S Babur C Özlem MB Investigation of Coxiella burnetii antibodies in sheep in Aydin region, Turkey Rev Med Vet 2005 156 336 340

[B12] Kennerman E Rousset E Gölcü E Dufour P Seroprevalence of Q fever (coxiellosis) in sheep from the Southern Marmara Region, Turkey Comp Immunol Microbiol Infect Dis 2010 33 37 45 10.1016/j.cimid.2008.07.007 18848356

[B13] Ruiz-Fons F Astobiza I Barandika JF Hurtado A Atxaerandio R Juste RA García-Pérez AL Seroepidemiological study of Q fever in domestic ruminants in semi-extensive grazing systems BMC Vet Res 2010 6 3 10.1186/1746-6148-6-3 20089188 PMC2831013

[B14] Rodríguez NF Carranza C Bolaños M Pérez-Arellano JL Gutierrez C Seroprevalence of *Coxiella burneti *in domestic ruminants in Gran Canaria Island, Spain Transbound Emerg Dis 2010 57 66 67 10.1111/j.1865-1682.2010.01116.x 20537108

[B15] Capuano F Parisi A Cafiero MA Pitaro L Fenizia D *Coxiella burneti*: what is the reality? Parassitologia 2004 46 131 134 15305702

[B16] Serbezov VS Kazár J Novkirishki V Gatcheva N Kovácová E Voynova V Q fever in Bulgaria and Slovakia Emerg Infect Dis 1999 5 388 394 10.3201/eid0503.990309 10341175 PMC2640784

[B17] Guatteo R Beaudeau F Berri M Rodolakis A Joly A Seegers H Shedding routes of *Coxiella burneti *in dairy cows: implications for detection and control Vet Res 2006 37 827 833 10.1051/vetres:2006038 16973121

[B18] Rousset E Durand B Berri M Dufour P Prigent M Russo P Delcroix T Touratier A Rodolakis A Aubert M Comparative diagnostic potential of three serological tests for abortive Q fever in goat herds Vet Microbiol 2007 124 286 297 10.1016/j.vetmic.2007.04.033 17532581

[B19] Rodolakis A Berri M Héchard C Caudron C Souriau A Bodier CC Blanchard B Camuset P Devillechaise P Natorp JC Vadet JP Arricau-Bouvery N Comparison of *Coxiella burneti *shedding in milk of dairy bovine, caprine, and ovine herds J Dairy Sci 2007 90 5352 5360 10.3168/jds.2006-815 18024725

[B20] Berri M Souriau A Crosby M Rodolakis A Shedding of *Coxiella burneti *in ewes in two pregnancies following an episode of Coxiella abortion in a sheep flock Vet Microbiol 2002 85 55 60 10.1016/S0378-1135(01)00480-1 11792492

[B21] Oporto B Barandika JF Hurtado A Aduriz G Moreno B Garcia-Perez AL Incidence of ovine abortion by *Coxiella burneti *in northern Spain Ann N Y Acad Sci 2006 1078 498 501 10.1196/annals.1374.095 17114763

[B22] Parisi A Fraccalvieri R Cafiero M Miccolupo A Padalino I Montagna C Capuano F Sottili R Diagnosis of *Coxiella burneti*-related abortion in Italian domestic ruminants using single-tube nested PCR Vet Microbiol 2006 118 101 106 10.1016/j.vetmic.2006.06.023 16891064

[B23] Masala G Porcu R Sanna G Tanda A Tola S Role of *Chlamydophila abortu *in ovine and caprine abortion in Sardinia, Italy Vet Res Commun 2005 29 117 123 15943071 10.1007/s11259-005-0842-2

[B24] Clemente L Barahona MJ Andrade MF Botelho A Diagnosis by PCR of *Coxiella burneti *in aborted fetuses of domestic ruminants in Portugal Vet Rec 2009 164 373 374 10.1136/vr.164.12.373 19305010

[B25] Öngör H Cetinkaya B Karahan M Açik MN Bulut H Muz A Detection of *Coxiella burneti *by immunomagnetic separation-PCR in the milk of sheep in Turkey Vet Rec 2004 154 570 572 10.1136/vr.154.18.570 15144006

[B26] Samuel JE Frazier ME Kahn ML Thomashow LS Mallavia LP Isolation and characterization of a plasmid from phase I *Coxiella burneti* Infect Immun 1983 41 488 493 6307871 10.1128/iai.41.2.488-493.1983 PMC264667

[B27] Willems H Thiele D Krauss H Plasmid based differentiation and detection of *Coxiella burneti *in clinical samples Eur J Epidemiol 1993 9 411 418 10.1007/BF00157399 8243597

[B28] Porten K Rissland J Tigges A Broll S Hopp W Lunemann M van Treeck U Kimmig P Brockmann SO Wagner-Wiening C Hellenbrand W Buchholz U A super-spreading ewe infects hundreds with Q fever at a farmers' market in Germany BMC Infect Dis 2006 6 147 10.1186/1471-2334-6-147 17026751 PMC1618839

[B29] Klaassen CH Nabuurs-Franssen MH Tilburg JJ Hamans MA Horrevorts AM Multigenotype Q fever outbreak, the Netherlands Emerg Infect Dis 2009 15 613 614 10.3201/eid1504.081612 19331749 PMC2671457

[B30] Roest HI Ruuls RC Tilburg JJ Nabuurs-Franssen MH Klaassen CH Vellema P van den Brom R Dercksen D Wouda W Spierenburg MA van der Spek AN Buijs R de Boer AG Willemsen PT van Zijderveld FG Molecular epidemiology of Coxiella burnetii from ruminants in Q fever outbreak, the Netherlands Emerg Infect Dis 2011 17 668 675 21470457 10.3201/eid1704.101562 PMC3377418

[B31] Enright JB Franti CE Longhurst WM Behymer DE Wright ME Dutson VJ *Coxiella burneti *in a wildlife-livestock environment. Antibody response of ewes and lambs in an endemic Q fever area Am J Epidemiol 1971 94 62 71 5105320 10.1093/oxfordjournals.aje.a121296

[B32] Arricau-Bouvery N Hauck Y Bejaoui A Frangoulidis D Bodier CC Souriau A Meyer H Neubauer H Rodolakis A Vergnaud G Molecular characterization of *Coxiella burneti *isolates by infrequent restriction site-PCR and MLVA typing BMC Microbiol 2006 6 38 10.1186/1471-2180-6-38 16640773 PMC1488860

[B33] Cannon RM Roe TM Livestock disease surveys: a field manual for veterinarians 1982 Canberra: Australian Government Publishing Service

[B34] Programm zur Förderung der Tiergesundheit in den Schaf- und Ziegenbeständen in Thüringen Thüringer Staatsanzeiger 2008 16 564 565

[B35] Giménez DF Staining *Rickettsia *in yolk-sac cultures Stain Technol 1964 39 135 140 14157454 10.3109/10520296409061219

[B36] Zhang GQ Hotta A Mizutani M Ho T Yamaguchi T Fukushi H Hirai K Direct identification of *Coxiella burneti *plasmids in human sera by nested PCR J Clin Microbiol 1998 36 2210 2213 9665993 10.1128/jcm.36.8.2210-2213.1998 PMC105014

[B37] Klee SR Tyczka J Ellerbrok H Franz T Linke S Baljer G Appel B Highly sensitive real-time PCR for specific detection and quantification of *Coxiella burneti* BMC Microbiol 2006 6 2 10.1186/1471-2180-6-2 16423303 PMC1360083

